# Oculomics in Gastrointestinal Disorders: A narrative review

**DOI:** 10.22336/rjo.2026.51

**Published:** 2026

**Authors:** Arvind Kumar Morya, Srishti Khullar, Hemlata Udenia, Puja Hingorani-Bang, Shweta Walia, Prince Manchanda, Anagha Shanavas, Rutuja Nagare

**Affiliations:** 1 Pediatric Ophthalmology and Medical Retina Services, All India Institute of Medical Sciences, Hyderabad, Telangana, India; 2 Military Hospital, Agra, Uttar Pradesh, India; 3 Dr. Rajendra Prasad Centre for Ophthalmic Sciences, AIIMS, New Delhi, India; 4 Department of Ophthalmology, AIIMS, Nagpur, Maharashtra, India; 5 Department of Ophthalmology, MGM Medical College, Indore, Madhya Pradesh, India; 6 Department of Internal Medicine, Military Hospital, Agra Cantt, Agra, Uttar Pradesh, India; 7 Regional Institute of Ophthalmology (RIO), Institute of Medical Sciences, Banaras Hindu University, Varanasi, Uttar Pradesh, India; 8 Department of Ophthalmology, Sri Sankara Netra Chikitsalaya, Eluru, West Godavari Di, Andhra Pradesh, India

**Keywords:** oculomics, gastrointestinal disease, artificial intelligence, retinal imaging, ocular biomarkers, AI = artificial intelligence, AUC = area under the curve, CNN = convolutional neural network, FAZ = foveal avascular zone, GI = gastrointestinal, IBD = inflammatory bowel disease, MASLD = metabolic dysfunction-associated steatotic liver disease, ML = machine learning, NAFLD = non-alcoholic fatty liver disease, OCT = optical coherence tomography, OCTA = optical coherence tomography angiography, RNFL = retinal nerve fibre layer

## Abstract

**Aim:**

We reviewed various ocular biomarkers relevant to gastrointestinal (GI) disorders and examined the potential role of artificial intelligence (AI) in detecting, integrating, and translating these biomarkers into clinical gastroenterology practice.

**Methods:**

A literature search from PubMed, MEDLINE, Scopus, Google Scholar, and Web of Science from the year 2000 up to February 2026 was conducted. We included published articles on ocular findings in gastrointestinal disorders and/or AI-based analysis of ocular biomarkers with gastrointestinal connections. Results suggested a positive correlation between AI-based ocular biomarkers and GI disorders.

**Results:**

After reviewing the literature, we found that the most frequently described ocular biomarkers were retinal vascular caliber and arteriovenous ratio on fundus photography; retinal nerve fibre layer and choroidal changes on optical coherence tomography; and vessel-density changes on optical coherence tomography angiography. The persistent signal came from inflammatory bowel disease and steatotic liver disease. AI may improve image processing, feature extraction, and pattern recognition, but more multicentric studies are needed to establish definitive analogy.

**Discussion:**

This review suggests that ocular biomarkers could provide a simple, non-invasive way to identify and monitor gastrointestinal diseases, especially inflammatory bowel disease and steatotic liver disease. Changes in retinal blood vessels, the retinal nerve fibre layer, the choroid, and OCTA-derived vessel density may reflect the inflammation, metabolic disturbances, and small-vessel changes associated with these conditions. However, these findings should be interpreted with caution because many existing studies included small or selected patient groups, used cross-sectional designs, applied different imaging methods, and did not consistently account for factors such as age, vascular risk, medications, or coexisting eye disease. Artificial intelligence may improve the analysis of complex eye images by identifying subtle patterns. Still, high model performance alone does not prove that these tools are accurate, reliable, or specific enough for clinical use. Future studies should therefore include large, multicentre, prospective populations, standardized imaging and reporting methods, independent external validation, and clinically meaningful outcomes. These steps will help determine whether AI-assisted oculomics can reliably support gastrointestinal screening, disease classification, risk assessment, and long-term monitoring.

**Conclusions:**

Various ocular biomarkers offer a promising, non-invasive approach to studying the deeper nuances of gastrointestinal disease. AI may help scale early biomarker detection and avoid gaps in analysis. Still, routine clinical use will require better external validation, clearer reporting standards, and prospective translational studies.

## Introduction

Oculomics uses ocular findings to investigate systemic health and disease. The eye offers a rare opportunity to examine neural tissue, microvasculature, and external adnexal structures without invasive testing, which makes ophthalmic imaging attractive for systemic biomarker research. Slit-lamp examination, external-eye and fundus photography, optical coherence tomography (OCT), OCT angiography (OCTA), and ocular-fluid analysis can all generate quantitative data relevant to screening, early detection, risk stratification, prognosis, and treatment monitoring [1-6]. AI methods, especially machine learning and deep learning, have widened the scale at which these multimodal data can be analyzed [1-6]. Although the field is advancing quickly, a focused review of AI-enabled ocular biomarkers across gastrointestinal (GI) disorders remains absent; most dedicated syntheses to date have focused on hepatobiliary disease [7]. Therefore, this review brings together the available evidence across hepatobiliary disorders, inflammatory bowel disease, and other GI conditions and considers how AI might support clinical translation.

The eye is uniquely suited to systemic biomarker research because it offers direct, repeatable, and relatively low-cost access to both the microcirculation and the central nervous system. Retinal vessels are anatomically and physiologically analogous to cerebral and systemic microvasculature. At the same time, the retina, choroid, cornea, tear film, lens, and ocular adnexa each provide distinct forms of structural, vascular, neural, inflammatory, or biochemical information [2-6]. Recent oculomics studies have shown that ocular images may encode not only ophthalmic disease but also age, sex, smoking exposure, blood pressure, cardiometabolic risk, and broader systemic phenotypes [1-6]. Importantly, the value of ocular biomarkers is not necessarily tied to disease specificity at the level of a single feature. Rather, the future of oculomics lies in integrating multimodal signals across ocular structures and combining them with AI-enabled pattern recognition to improve specificity, support differential diagnosis, and enable clinically usable prediction models [1,4-6].

A biologically plausible gut-liver-eye axis provides the conceptual basis for studying ocular biomarkers in GI disease. The gut microbiome can influence distant organs through inflammatory, immune, neuroendocrine, and metabolic pathways, and dysbiosis may disrupt epithelial and vascular barriers, promote oxidative stress, and alter host immune regulation [8-11]. These mechanisms are relevant to ophthalmology because microbial and systemic inflammatory signals have been linked to dry eye disease, uveitis, retinal vascular dysfunction, glaucoma, and other ocular disorders [8-11]. In parallel, liver disease can alter systemic metabolism, lipid handling, vitamin storage, bilirubin metabolism, and endothelial function; these changes may be reflected in the conjunctiva, sclera, cornea, retina, and choroid. Therefore, the eye may serve as a downstream readout of gut-derived inflammation, liver-related metabolic dysfunction, or both.

Dynamic, fast-evolving AI-based ocular biomarker applications in gastroenterology can help with screening, triage, phenotyping, and follow-ups. AI-assisted retinal screening platforms have already demonstrated strong performance in ophthalmology and systemic-risk prediction, illustrating how scalable ocular image analysis can be deployed in real-world workflows [12]. In hepatobiliary disease, deep-learning models trained on external-eye or fundus images have shown promising discriminatory performance for cirrhosis, liver cancer, and other hepatobiliary conditions. At the same time, smartphone-based applications for scleral jaundice have demonstrated potential for low-cost bilirubin assessment and home monitoring [13,14]. These examples do not yet establish routine GI practice, but they strongly support the feasibility of ocular imaging as a scalable adjunctive biomarker platform for chronic GI disease.

## Methods

### Review design

We conducted a narrative review to study the published literature on Artificial Intelligence (AI)-based detection of ocular biomarkers in gastrointestinal disorders.

### Literature search strategy

We conducted an extensive literature search using PubMed/MEDLINE, Scopus, and Web of Science for articles published from 2000 to February 2026. Keywords and MeSH terms included “ocular biomarkers”, “retinal imaging”, “artificial intelligence”, “deep learning”, “machine learning”, “gastrointestinal disorders”, “inflammatory bowel disease”, “celiac disease”, “liver disease”, “optical coherence tomography”, “fundus photography”, and “retinal vasculature”, using Boolean operators (AND/OR).

### Eligibility criteria

We included only published studies with ocular findings in humans and animals and confirmed gastrointestinal disorders. We excluded abstracts, preprints, editorials, and opinion articles without primary data.

### Study selection and data extraction

Two reviewers independently screened articles for relevance, with a third resolving disagreements. We recorded study design, demographic characteristics, gastrointestinal condition, ocular parameters, imaging modality, and AI details.

### Data synthesis plan

We arranged findings thematically into structural, vascular, and functional ocular changes; AI frameworks; and disease-specific associations.

## Results

### Overview of Included Evidence

Most of the literature identified for this review consisted of observational or cross-sectional studies examining retinal or ocular-surface features in patients with hepatobiliary disease or inflammatory bowel disease [15,16]. These studies mainly explored associations rather than validated clinical tests. Parallel advances in retinal AI, illustrated by large-image analyses from Poplin and Ting, have shown that fundus photographs can contain subtle vascular and structural signals linked to systemic disease [17,18].

### Ocular Imaging Modalities and Candidate Biomarkers

Fundus photography, optical coherence tomography (OCT), and optical coherence tomography angiography (OCTA) are the main ocular imaging tools used to investigate GI-related biomarkers.

Fundus photography enables high-resolution imaging of retinal vasculature and optic disc architecture, and is widely employed to assess retinal vascular parameters associated with systemic diseases [19]. OCT provides cross-sectional imaging of retinal layers and the choroid, enabling the detection of subtle structural changes associated with neurodegenerative or inflammatory disorders [20]. As a novel technique, OCTA facilitates non-invasive assessment of retinal microvascular perfusion and capillary density [21].

### Fundus Photography Markers

Fundus photography is one of the most widely used imaging modalities in ocular biomarker research because of its accessibility, low cost, and compatibility with automated image analysis. Among fundus-based indicators, retinal vascular caliber is frequently reported. Changes in the diameter of retinal arterioles and venules have been associated with systemic inflammatory and metabolic disorders [19,22]. Similarly, alterations in the retinal arteriovenous ratio, particularly arteriolar narrowing and venular dilation, have been linked to metabolic syndrome and systemic inflammation, conditions that may coexist with gastrointestinal diseases [23].

**Fig. 1:**
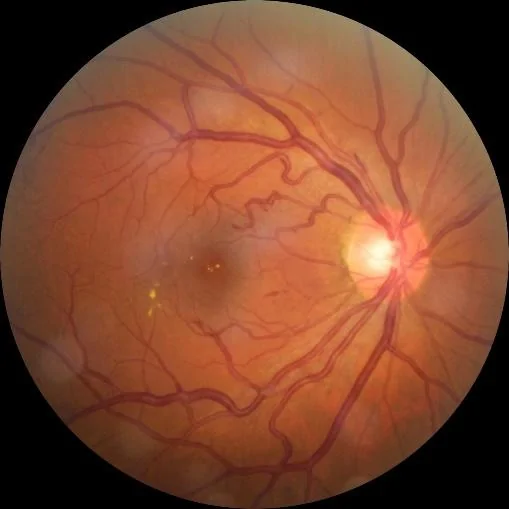
Arteriovenous changes in a patient with chronic IBD

Additional retinal features include vascular tortuosity, branching patterns, and optic disc morphology. AI-driven analysis enables automated quantification of these characteristics and improves the detection of subtle vascular abnormalities. Deep learning models can predict systemic conditions such as cardiovascular and metabolic diseases from fundus photographs alone, as shown in studies by Poplin et al. [18]. However, further validation is needed because these applications have a limited role in gastrointestinal diseases.

### OCT-Derived Biomarkers in GI Disorders

Several studies have reported thinning in the retinal nerve fibre layer (RNFL) and related macular alterations in patients with inflammatory bowel disease, including Crohn’s disease and ulcerative colitis. These variables may reflect possible interplay between chronic systemic inflammation and neuroinflammatory processes or microvascular compromise [24].

Studies evaluating patients with metabolic and hepatic diseases, such as non-alcoholic fatty liver disease, have reported alterations in another parameter known as subfoveal choroidal thickness [25]. These changes may reflect systemic vascular dysregulation, inflammatory cytokine activity, or metabolic disturbances affecting choroidal circulation.

Changes in macular thickness and retinal layer integrity have also been described in inflammatory bowel disease and other chronic inflammatory states [26]. Advances in AI-assisted segmentation techniques now allow automated analysis of retinal layer thickness across OCT datasets, enabling detection of subtle structural variations that may serve as systemic disease biomarkers.

Overall, OCT-derived parameters provide valuable structural insights into retinal and choroidal changes that may reflect systemic inflammatory and metabolic processes associated with gastrointestinal diseases.

### OCTA Microvascular Biomarkers

OCTA enables non-invasive imaging of retinal and choroidal microvasculature without contrast agents. Various OCTA studies have suggested reduced retinal vessel density in the superficial and deep capillary plexuses among patients with inflammatory bowel disease [26]. This suggests chronic inflammation driven by microvascular injury and systemic endothelial dysfunction.

Alterations in the foveal avascular zone (FAZ) have been verified in systemic inflammatory and metabolic disorders, indicating impaired retinal perfusion and disrupted vascular autoregulation [21]. Furthermore, optic nerve microcirculation decreases because peripapillary vessel density declines.

AI applications can analyze OCTA images to automatically measure vessel density, perfusion indices, and capillary non-perfusion regions, improving detection of subtle vascular abnormalities. Despite the limited number of OCTA-based studies focused on gastrointestinal diseases, this technique shows potential for detecting systemic microvascular changes evident in retinal circulation.

### Anterior Segment and Ocular Surface Biomarkers

Beyond retinal imaging findings, several gastrointestinal diseases are associated with ocular surface and anterior segment manifestations that may indicate systemic inflammatory activity.

Ocular surface abnormalities such as dry eye symptoms, conjunctival hyperemia, and tear film instability have been reported in patients with inflammatory bowel disease [15]. Chronic immune activation and inflammatory cytokine release may contribute to lacrimal gland dysfunction and ocular surface inflammation.

Patients with Crohn’s disease and ulcerative colitis may also develop episcleritis, scleritis, and anterior uveitis, which are recognized extraintestinal manifestations of these diseases [15,16]. Episcleritis often correlates with active intestinal inflammation, whereas uveitis may occur independently of gastrointestinal disease activity.

Furthermore, corneal nerve fibre alterations, visualized using in vivo confocal microscopy, have been investigated as potential biomarkers of systemic neuropathy and inflammatory activity [26,27]. Although evidence specifically linking corneal nerve changes to gastrointestinal diseases remains limited, such markers may provide insights into systemic neuroinflammatory mechanisms.

Overall, integrating anterior- and posterior-segment ocular findings may provide a comprehensive framework for AI-based identification of systemic biomarkers associated with gastrointestinal disorders. **Table 1** illustrates the interconnected trajectories between GI disorders and ocular biomarkers. **Table 1**. Conceptual overview of candidate ocular biomarkers across gastrointestinal disorders

**Table 1:** Conceptual overview of candidate ocular biomarkers across gastrointestinal disorders

Gastrointestinal disorder	Ocular imaging modality	Reported ocular biomarkers	Key findings
Metabolic dysfunction-associated steatotic liver disease / nonalcoholic steatohepatitis	Optical coherence tomography	Increased or altered choroidal thickness; retinal structural changes	Ocular microvascular alterations may reflect metabolic and inflammatory processes associated with hepatic steatosis.
Liver cirrhosis	Fundus photography	Retinal vascular abnormalities; vessel-caliber changes	Systemic hemodynamic alterations and endothelial dysfunction may influence retinal microcirculation.
Inflammatory bowel disease	Optical coherence tomography	Retinal nerve fiber layer thickness changes; macular alterations	Retinal structural changes may reflect systemic inflammatory activity.
Crohn disease / ulcerative colitis	Optical coherence tomography angiography	Reduced retinal capillary density; altered foveal avascular zone	Microvascular changes may reflect chronic systemic inflammation.
Celiac disease	Fundus photography / ocular surface evaluation	Ocular surface abnormalities, retinal dysfunction related to vitamin deficiency	Nutritional deficiencies secondary to malabsorption may affect ocular tissues.
Pancreatitis / pancreaticobiliary disorders	Optical coherence tomography / OCT angiography	Retinal vascular alterations	Systemic inflammatory and vascular effects may influence retinal circulation.
Colorectal cancer	Fundus photography	Retinal microvascular changes	Systemic metabolic and inflammatory alterations may be reflected in retinal vasculature.

### AI Methods for Ocular Biomarker Detection

Integrating AI with retinal imaging modalities such as Fundus photography, OCT, and OCTA has enabled automated extraction of clinically relevant features that may reflect systemic inflammatory, metabolic, or vascular alterations associated with gastrointestinal disorders. Machine learning and deep learning advancements have significantly improved the accuracy and scalability of ocular image analysis for biomarker discovery [17,28-31].

### Traditional Machine Learning (ML) versus Deep Learning (DL)

Traditional ML requires predefined feature selection and domain expertise, which constrains its application. Whereas deep learning approaches, particularly convolutional neural networks (CNNs), allow automatic extraction of advanced image features directly from raw data. CNN-based models can detect intricate patterns within retinal images that may not be easily identifiable through conventional ML methods [17,18,28].

DL algorithms have shown high diagnostic accuracy in multiple ocular applications, including detecting diabetic retinopathy, age-related macular degeneration, glaucoma, and other retinal diseases. Several landmark studies have shown that DL models trained on large retinal imaging datasets can achieve sensitivity and specificity comparable to, or better than, expert ophthalmologists in biomarker detection tasks [30-32]. Ample evidence supports the superiority of deep learning systems for identifying novel ocular biomarkers in many systemic disease processes.

### Model Architectures and Training Strategies

Numerous DL models have been applied in ocular image analysis. The most widely used architecture for retinal image classification is the convolutional neural network (CNN) because it can capture multi-dimensional relationships within images. Common CNN architectures used in medical image analysis include deep residual networks (ResNet), VGG networks, and Inception-based models [32,33].

For further evaluation of ocular images, segmentation architectures such as U-Net have been widely applied to delineate retinal layers and vascular structures in OCT and fundus images. Segmentation methodologies can support in-depth quantitative analysis of ocular biomarkers such as retinal thickness, vessel density, and capillary morphology [34].

Supervised learning requires annotated imaging datasets to train AI models. This process requires large, labeled datasets to develop robust, generalizable AI applications. To address limitations in dataset size, researchers often apply data augmentation techniques such as image rotation, flipping, scaling, and contrast adjustment to increase training diversity and reduce overfitting [17].

To improve transparency in DL models, researchers have developed several explainable AI techniques. Visualization tools such as saliency maps and gradient-weighted class activation mapping (Grad-CAM) identify image regions that most strongly influence model predictions [35]. These visualization approaches enable researchers and clinicians to determine whether AI models focus on clinically relevant anatomical structures in retinal images.

In ophthalmology, AI methods have shown that deep learning models rely on retinal vascular features and optic disc characteristics when predicting systemic cardiovascular risk factors from fundus photographs [18]. Such findings suggest that similar AI-based approaches could potentially identify retinal biomarkers associated with systemic inflammatory or metabolic diseases.

### Validation and Performance Metrics

AI models in ocular imaging require validation, commonly evaluated using standard tests such as accuracy, sensitivity, specificity, precision, and F1 score. The area under the receiver operating characteristic curve (AUC) is used as an overall measure of a model’s ascendancy in many diagnostic studies [17].

Cross-validation and calibration analysis are also needed to assess model stability and reliability. Such validation is critical for AI systems developed to identify systemic disease biomarkers from ocular images, where reproducibility and clear clinical interpretation are essential [36-38].

### Evidence by GI Disorder Category

A targeted literature search suggests that GI disorders may involve retinal, choroidal, or ocular-surface changes. Although evidence is stronger for hepatobiliary disease and inflammatory bowel disease, evidence for other GI disorders remains limited and often exploratory because fewer studies exist.

### Hepatobiliary Disorders

Hepatobiliary disorders are among the best-studied GI conditions in ocular biomarker research. Steatotic liver disease, steatohepatitis, hepatitis, and cirrhosis share metabolic, inflammatory, and vascular mechanisms that may affect ocular microcirculation. Reported findings include altered subfoveal choroidal thickness in fatty liver disease [25] and broader vascular changes that may parallel systemic endothelial dysfunction [22]. In advanced liver disease, ocular findings are interpreted as downstream reflections of circulatory and metabolic disturbance rather than as disease-specific markers [39]. Cholestatic disease may also produce visible ocular-surface or scleral changes through bilirubin deposition. Overall, the signal is biologically probable, but the clinical meaning of these findings still needs validation.

### Inflammatory Bowel Disease

Ocular involvement is well recognized in patients with inflammatory bowel disease, more so in Crohn’s disease and ulcerative colitis. Ocular manifestations are considered among the most common extraintestinal complications of these disorders and may occur in up to 10% of patients [15].

Anterior segment inflammatory conditions such as episcleritis, scleritis, and uveitis are frequently reported in IBD. Uveitis is found independently of gastrointestinal symptoms, whereas episcleritis is more often observed with active intestinal disease [15]. The immune-mediated mechanisms and systemic cytokine dysregulation lead to ocular inflammation.

Recent studies have used optical coherence tomography in IBD to investigate retinal structural changes alongside clinical findings. Confirmed reports of alterations in retinal nerve fibre layer density and macular thickness suggest possible neuroinflammatory involvement of retinal tissues [39].

To take it further, Optical Coherence Tomography Angiography is a valuable tool for assessing subtle microvascular changes. In IBD, systemic endothelial dysfunction and chronic inflammation may reduce retinal capillary density and alter the foveal avascular zone [26].

These studies suggest that retinal microvascular change could serve as an adjunctive ocular biomarker of systemic inflammatory activity in IBD; however, current evidence is too limited, and more studies are needed to support its applicability in routine clinical use.

### Other Gastrointestinal Conditions

Other than hepatobiliary disorders and IBD, patients with Celiac disease and other malabsorption syndromes may develop ocular manifestations due to nutritional deficiencies. Vitamin A, D, and B-complex nutrient deficiencies can lead to ocular surface abnormalities, night blindness, or retinal dysfunction [40]. In these conditions, ocular findings may represent secondary effects of systemic nutritional deficits.

The literature has occasionally associated pancreatitis with ocular complications such as retinal vascular occlusions or inflammatory changes. Although these associations are relatively rare, they may help link GI disorders to ocular tissues through systemic vascular and inflammatory pathways.

Evidence for ocular biomarkers in GI malignancy is far less developed than in hepatobiliary disease or IBD. Any link between colorectal cancer and retinal microvascular change remains preliminary and should be interpreted cautiously [41]. Ocular imaging may eventually help detect systemic effects of malignancy, but at present, this area is better viewed as hypothesis-generating than practice-changing. Overall, ocular imaging offers a non-invasive window into systemic pathophysiology across several GI disorders, but evidence maturity differs markedly by disease group. **Table 2** summarizes the major biomarkers and imaging modalities. **Table 2**. Representative studies and imaging modalities relevant to ocular biomarkers in gastrointestinal disorders

**Table 2:** Representative studies and imaging modalities relevant to ocular biomarkers in gastrointestinal disorders

GI disorder	Ocular imaging modality	Candidate ocular biomarker	Key findings	Representative studies
Inflammatory bowel disease	Fundus photography	Retinal vascular caliber changes; arteriovenous ratio	Altered retinal microvascular parameters reflecting systemic inflammation and vascular dysfunction	Wong et al.; Cheung et al.
Inflammatory bowel disease	Optical coherence tomography	RNFL thickness; macular thickness	Subtle retinal neuronal-layer changes associated with chronic systemic inflammation	Coskun et al.
Inflammatory bowel disease	Optical coherence tomography angiography	Vessel density; foveal avascular zone	Reduced capillary density suggesting systemic microvascular impairment	Migliorisi et al.
Crohn disease	Clinical anterior segment evaluation	Episcleritis, uveitis	Extraintestinal inflammatory manifestations correlated with systemic disease activity	Mintz et al.
Ulcerative colitis	Ocular surface imaging	Dry-eye parameters: tear-film instability	Increased prevalence of ocular surface disease in IBD patients	Mintz et al.; Vavricka et al.
Non-alcoholic fatty liver disease / MASLD	Optical coherence tomography	Choroidal thickness	Altered choroidal circulation linked to metabolic and vascular change	Avci et al.
Liver cirrhosis	Fundus photography	Retinal vascular abnormalities	Microvascular changes associated with systemic circulatory alterations	Iwakiri and Groszmann
Systemic inflammatory GI diseases	In vivo confocal microscopy	Corneal nerve fiber density	Reduced corneal nerve density may reflect systemic inflammation and neuropathy.	Petropoulos et al.; Tavakoli et al.
Multiple GI disorders	AI-based retinal image analysis	Deep-learning retinal features	AI models can detect systemic disease patterns from retinal images.	Poplin et al.; Ting et al.

### Correlation with Clinical Phenotypes and Outcomes

Ocular imaging biomarkers may also have value beyond disease detection. Because the retina and choroid share vascular and immunologic features with other organ systems, ocular changes may track clinically meaningful phenotypes such as disease burden, inflammatory activity, treatment response, and long-term vascular risk in GI disorders.

### Diagnosis, Screening, and Triage

Ocular biomarkers may have a role in screening and triage because retinal vascular features can reflect broader metabolic and inflammatory states [42]. Fundus-derived measures such as vessel caliber, tortuosity, and branching pattern have already been linked to hypertension, diabetes, and metabolic syndrome [43-45]. AI-based retinal analysis extends this concept by extracting systemic risk information directly from images [18]. For GI disease, the likely early use case is not a standalone diagnosis but rapid risk enrichment or supportive triage in settings where more complex testing is limited.

### Severity and Staging of Disease

In selected GI disorders, ocular findings may also correlate with disease severity. Structural retinal and choroidal changes can reflect the burden of systemic metabolic or inflammatory stress [19]. In steatotic liver disease, for example, ocular vascular or choroidal abnormalities may parallel the underlying metabolic milieu rather than act as a direct surrogate for liver histology [25,44]. Quantitative OCT and OCTA measurements, therefore, remain promising, but their role in staging disease is not yet settled.

### Pathological Activity and Therapeutic Response

Another area of interest is whether ocular biomarkers change with disease activity or treatment response. In IBD, ocular inflammation may accompany intestinal activity, and newer imaging studies suggest that retinal microvascular parameters may vary with systemic inflammatory burden [15,26]. If confirmed in longitudinal studies, AI-assisted image analysis could help detect subtle interval change and support non-invasive monitoring over time.

### Prognosis and Complications

Ocular biomarkers may also provide prognostic information by reflecting the broader vascular risk carried by chronic GI disease. Retinal microvascular abnormalities are already associated with cardiovascular events, stroke, and mortality in population studies [22]. The current evidence suggests that ocular vascular features may add context to long-term complication risk. However, they remain an adjunctive rather than established prognostic tool for GI conditions linked to systemic inflammation or metabolic dysfunction.

## Discussion

In this first review, the literature supports the concept that ocular biomarkers may serve as a non-invasive screening and diagnostic tool for GI disease, especially in disorders characterized by chronic inflammation, endothelial dysfunction, immune activation, and metabolic stress.

The retina is a metabolically active neurovascular tissue with dense microcirculation and distinct structural layers, so it may reflect systemic vascular and inflammatory injury in ways that can be imaged non-invasively. In IBD, ocular disease is already recognized as part of the extraintestinal spectrum, and newer imaging studies suggest that posterior-segment changes may extend beyond overt ocular inflammation [45-47]. In steatotic liver disease, chronic low-grade inflammation, insulin resistance, dyslipidemia, and endothelial dysfunction provide a plausible basis for retinal and choroidal change [44,45,48,49]. Celiac disease offers a different model, in which ocular findings may arise from a combination of immune disturbance, nutritional deficiency, and microvascular injury [15].

Across the literature reviewed, the most consistent ocular signals were observed in IBD and steatotic liver disease. The principal biomarkers included retinal vascular caliber and arteriovenous ratio on fundus photography, retinal nerve fiber layer and choroidal parameters on OCT, and retinal vessel density measures on OCTA. These findings support the concept that the eye may provide a non-invasive window into systemic inflammatory, metabolic, and vascular processes associated with GI disease. However, heterogeneity in study populations, imaging protocols, biomarker definitions, and analytical methods limit direct comparison across studies. Accordingly, these ocular features should currently be considered candidate biomarkers rather than established diagnostic markers. AI may enhance their utility through automated feature extraction and recognition of subtle patterns, but their clinical relevance remains dependent on external validation and prospective evaluation.

Clinically, AI-based ocular biomarkers are likely to support, rather than define, GI disease management. Retinal imaging alone may not confirm IBD. They must be well correlated with laboratory markers, endoscopy, and cross-sectional imaging by providing a rapid, repeatable view of systemic inflammatory or vascular burden [46]. In short, ocular biomarkers in steatotic liver disease are value-added only and far beyond established non-invasive tools such as fibrosis scores and elastography. Hence, a realistic approach is to prioritize broader vascular risk stratification over liver-specific diagnosis, as some retinal associations appear linked to obesity and other cardiometabolic factors [50].

The current evidence base remains constrained by several methodological weaknesses. Confounding is a major concern, especially for MASLD, cirrhosis, and pancreaticobiliary disease, because diabetes, hypertension, obesity, dyslipidemia, smoking exposure, and renal dysfunction independently alter retinal structure and microvasculature. An apparent gastrointestinal ocular biomarker may, in some studies, be a cardiometabolic biomarker in disguise. In a large 2025 cohort study on steatotic liver disease, the association with retinal vein occlusion weakened after adjustment for body mass index, suggesting that obesity may also drive the signal [50]. Studies also differ in imaging platforms, segmentation algorithms, scan density, preprocessing pipelines, and definitions of retinal thickness and OCTA-derived vascular metrics. Small sample sizes and cross-sectional designs further limit causal inference. The OCTA study in patients with acute pancreatitis showed early microvascular and retinal thickness differences during hospitalization. This is an important finding but remains limited proof of concept [51]. AI-specific bias adds complexity, as models trained on a specific population or device may struggle elsewhere because of domain shift, spectrum bias, and calibration drift. Heavy reliance on internal validation can lead to overestimated performance.

The next phase of this field should move beyond small single-modality studies toward multimodal, collaborative, and longitudinal designs. Multimodal fusion models that combine fundus photography, OCT, OCTA, ocular surface features, laboratory markers, fibrosis indices, anthropometry, inflammatory markers, and disease-specific clinical variables are likely to outperform image-only approaches. Longitudinal cohorts are equally important because the true clinical value of ocular biomarkers lies not simply in cross-sectional association but in whether they track prognostic factors over time. The OCT study in IBD patients offers an early example of this more clinically relevant direction by linking retinal and choroidal metrics to therapeutic response [47].

A major strength of this review is that it integrates three areas usually discussed separately: ocular biomarkers, gastrointestinal systemic disease, and AI-enabled image analysis. Another strength is that the discussion incorporates current PubMed-indexed literature through 2025, including recent work on inflammatory bowel disease, MASLD nomenclature and management, pancreatitis-associated retinal change, and contemporary AI reporting guidance [15,44-54]. The limitations of this review reflect those of the underlying evidence base, which is predominantly observational and often cross-sectional, with few GI-specific validations of AI models.

Future studies in this field should be designed and reported according to contemporary AI standards from the outset. TRIPOD+AI now provides updated guidance for clinical prediction model studies that use regression or machine-learning methods and replaces reliance on the original TRIPOD checklist [52]. CLAIM remains relevant for medical imaging because it improves transparency about data provenance, image acquisition, dataset partitioning, reference standards, preprocessing, and model evaluation [55]. For diagnostic accuracy studies, STARD-AI provides a dedicated reporting framework that is especially useful when comparing AI outputs with clinical reference standards in translational imaging research [56].

Overall, the available evidence supports the concept that ocular imaging may provide a noninvasive window into systemic inflammatory, metabolic, and vascular processes associated with GI diseases. Retinal vascular, structural, and choroidal parameters show recurring associations, particularly in IBD and steatotic liver disease, while AI may facilitate automated detection and integration of these signals. At present, however, these findings should be regarded as promising candidate biomarkers rather than established clinical diagnostic tools. Multicentric studies with standardized imaging protocols, appropriate adjustment for systemic confounders, external validation, and longitudinal clinical outcomes are needed before ocular biomarkers and AI-based analysis can be incorporated into routine GI practice.

## Conclusion

Ocular imaging biomarkers offer a promising non-invasive approach to studying GI disease, particularly in disorders driven by chronic inflammation, metabolic dysfunction, and microvascular injury. Fundus photography, OCT, and OCTA have identified candidate retinal, choroidal, and ocular-surface biomarkers in inflammatory bowel disease, hepatobiliary disease, and selected other GI conditions. Because the evidence remains heterogeneous and largely exploratory, AI may improve how these signals are detected and analyzed. For clinical use in GI disorders, more multimodal studies, external validation, longitudinal follow-up, and adherence to contemporary reporting standards will be essential.

## References

[1] Wang J, Wang YX, Zeng D, Zhu Z, Li D, Liu Y, Sheng B, Grzybowski A, Wong TY (2025). Artificial intelligence-enhanced retinal imaging as a biomarker for systemic diseases. Theranostics.

[2] Patterson EJ, Bounds AD, Wagner SK, Kadri-Langford R, Taylor R, Daly D (2024). Oculomics: A Crusade Against the Four Horsemen of Chronic Disease. Ophthalmol Ther.

[3] Tan YY, Kang HG, Lee CJ, Kim SS, Park S, Thakur S, Da Soh Z, Cho Y, Peng Q, Lee K, Tham YC, Rim TH, Cheng CY (2024). Prognostic potentials of AI in ophthalmology: systemic disease forecasting via retinal imaging. Eye Vis (Lond).

[4] Wagner SK, Fu DJ, Faes L, Liu X, Huemer J, Khalid H, Ferraz D, Korot E, Kelly C, Balaskas K, Denniston AK, Keane PA (2020). Insights into Systemic Disease through Retinal Imaging-Based Oculomics. Transl Vis Sci Technol.

[5] Wu JH, Liu TYA (2022). Application of Deep Learning to Retinal-Image-Based Oculomics for Evaluation of Systemic Health: A Review. J Clin Med.

[6] Babenko B, Traynis I, Chen C, Singh P, Uddin A, Cuadros J, Daskivich LP, Maa AY, Kim R, Kang EY, Matias Y, Corrado GS, Peng L, Webster DR, Semturs C, Krause J, Varadarajan AV, Hammel N, Liu Y (2023). A deep learning model for novel systemic biomarkers in photographs of the external eye: a retrospective study. Lancet Digit Health.

[7] Parmar UPS, Morya AK, Gupta PC, Arora A, Verma N (2025). Role of artificial intelligence-based ocular biomarkers in hepatobiliary diseases: A scoping review. World J Hepatol.

[8] Campagnoli LIM, Varesi A, Barbieri A, Marchesi N, Pascale A (2023). Targeting the Gut-Eye Axis: An Emerging Strategy to Face Ocular Diseases. Int J Mol Sci.

[9] Bai X, Xu Q, Zhang W, Wang C (2023). The Gut-Eye Axis: Correlation Between the Gut Microbiota and Autoimmune Dry Eye in Individuals With Sjögren Syndrome. Eye Contact Lens.

[10] Song J, Dong H, Wang T, Yu H, Yu J, Ma S, Song X, Sun Q, Xu Y, Liu M (2024). What is the impact of microbiota on dry eye: a literature review of the gut-eye axis. BMC Ophthalmol.

[11] Napolitano P, Filippelli M, Davinelli S, Bartollino S, dell'Omo R, Costagliola C (2021). Influence of gut microbiota on eye diseases: an overview. Ann Med.

[12] Popescu Patoni SI, Muşat AAM, Patoni C, Popescu MN, Munteanu M, Costache IB, Pîrvulescu RA, Mușat O (2023). Artificial intelligence in ophthalmology. Rom J Ophthalmol.

[13] Xiao W, Huang X, Wang JH, Lin DR, Zhu Y, Chen C (2021). Screening and identifying hepatobiliary diseases through deep learning using ocular images: a prospective, multicentre study. Lancet Digit Health.

[14] Park JH, Yang MJ, Kim JS, Park B, Kim JH, Sunwoo MH (2021). Deep-Learning-Based Smartphone Application for Self-Diagnosis of Scleral Jaundice in Patients with Hepatobiliary and Pancreatic Diseases. J Pers Med.

[15] Mintz R, Feller ER, Bahr RL, Shah SA (2004). Ocular manifestations of inflammatory bowel disease. Inflamm Bowel Dis.

[16] Vavricka SR, Schoepfer A, Scharl M, Lakatos PL, Navarini A, Rogler G (2015). Extraintestinal Manifestations of Inflammatory Bowel Disease. Inflamm Bowel Dis.

[17] Morya AK, Gowdar J, Kaushal A, Makwana N, Biswas S, Raj P, Singh S, Hegde S, Vaishnav R, Shetty S, S P V, Shah V, Paul S, Muralidhar S, Velis G, Padua W, Waghule T, Nazm N, Jeganathan S, Reddy Mallidi A, Susan John D, Sen S, Choudhary S, Parashar N, Sharma B, Raghav P, Udawat R, Ram S, Salodia UP (2021). Evaluating the Viability of a Smartphone-Based Annotation Tool for Faster and Accurate Image Labelling for Artificial Intelligence in Diabetic Retinopathy. Clin Ophthalmol.

[18] Poplin R, Varadarajan AV, Blumer K, Liu Y, McConnell MV, Corrado GS, Peng L, Webster DR (2018). Prediction of cardiovascular risk factors from retinal fundus photographs via deep learning. Nat Biomed Eng.

[19] Cheung CY, Ikram MK, Sabanayagam C, Wong TY (2012). Retinal microvasculature as a model to study the manifestations of hypertension. Hypertension.

[20] Spaide RF, Fujimoto JG, Waheed NK, Sadda SR, Staurenghi G (2018). Optical coherence tomography angiography. Prog Retin Eye Res.

[21] de Carlo TE, Romano A, Waheed NK, Duker JS (2015). A review of optical coherence tomography angiography (OCTA). Int J Retina Vitreous.

[22] Wong TY, Klein R, Klein BE, Tielsch JM, Hubbard L, Nieto FJ (2001). Retinal microvascular abnormalities and their relationship with hypertension, cardiovascular disease, and mortality. Surv Ophthalmol.

[23] Wong TY, Klein R, Sharrett AR, Duncan BB, Couper DJ, Tielsch JM, Klein BE, Hubbard LD (2002). Retinal arteriolar narrowing and risk of coronary heart disease in men and women. The Atherosclerosis Risk in Communities Study. JAMA.

[24] Ciotoracu AC, Dimăncescu MG, Mitulescu TC, Haralambie CI, Iorga AM, Busuioc C, Predețeanu D (2021). A clinical case of recurrent episcleritis as the initial manifestation of granulomatosis with polyangiitis. Rom J Ophthalmol.

[25] Avcı E, Kucukoduk A (2023). Choroidal vascular changes in non-alcoholic fatty liver disease. Endokrynol Pol.

[26] Coskun C, Coskun Y (2023). Evaluation of optic coherence tomography findings in patients with inflammatory bowel disease. Photodiagnosis Photodyn Ther.

[27] Petropoulos IN, Ponirakis G, Khan A, Gad H, Almuhannadi H, Brines M, Cerami A, Malik RA (2020). Corneal confocal microscopy: ready for prime time. Clin Exp Optom.

[28] Tavakoli M, Quattrini C, Abbott C, Kallinikos P, Marshall A, Finnigan J, Morgan P, Efron N, Boulton AJ, Malik RA (2010). Corneal confocal microscopy: a novel noninvasive test to diagnose and stratify the severity of human diabetic neuropathy. Diabetes Care.

[29] LeCun Y, Bengio Y, Hinton G (2015). Deep learning. Nature.

[30] Esteva A, Kuprel B, Novoa RA, Ko J, Swetter SM, Blau HM, Thrun S (2017). Dermatologist-level classification of skin cancer with deep neural networks. Nature.

[31] Gulshan V, Peng L, Coram M, Stumpe MC, Wu D, Narayanaswamy A, Venugopalan S, Widner K, Madams T, Cuadros J, Kim R, Raman R, Nelson PC, Mega JL, Webster DR (2016). Development and Validation of a Deep Learning Algorithm for Detection of Diabetic Retinopathy in Retinal Fundus Photographs. JAMA.

[32] De Fauw J, Ledsam JR, Romera-Paredes B, Nikolov S, Tomasev N, Blackwell S, Askham H, Glorot X, O'Donoghue B, Visentin D, van den Driessche G, Lakshminarayanan B, Meyer C, Mackinder F, Bouton S, Ayoub K, Chopra R, King D, Karthikesalingam A, Hughes CO, Raine R, Hughes J, Sim DA, Egan C, Tufail A, Montgomery H, Hassabis D, Rees G, Back T, Khaw PT, Suleyman M, Cornebise J, Keane PA, Ronneberger O (2018). Clinically applicable deep learning for diagnosis and referral in retinal disease. Nat Med.

[33] He K, Zhang X, Ren S, Sun J (2016). Deep residual learning for image recognition. Proceedings of the IEEE Conference on Computer Vision and Pattern Recognition (CVPR).

[34] Ronneberger O, Fischer P, Brox T (2015). U-Net: convolutional networks for biomedical image segmentation. arXiv [Preprint].

[35] Selvaraju RR, Cogswell M, Das A, Vedantam R, Parikh D, Batra D (2016). Grad-CAM: visual explanations from deep networks via gradient-based localization. arXiv [Preprint].

[36] Kelly CJ, Karthikesalingam A, Suleyman M, Corrado G, King D (2019). Key challenges for delivering clinical impact with artificial intelligence. BMC Med.

[37] Liu X, Cruz Rivera S, Moher D, Calvert MJ, Denniston AK, SPIRIT-AI and CONSORT-AI Working Group (2020). Reporting guidelines for clinical trial reports for interventions involving artificial intelligence: the CONSORT-AI extension. Lancet Digit Health.

[38] Beam AL, Kohane IS (2018). Big Data and Machine Learning in Health Care. JAMA.

[39] Iwakiri Y, Groszmann RJ (2007). Vascular endothelial dysfunction in cirrhosis. J Hepatol.

[40] Russell RM (2000). The vitamin A spectrum: from deficiency to toxicity. Am J Clin Nutr.

[41] Arnold M, Sierra MS, Laversanne M, Soerjomataram I, Jemal A, Bray F (2017). Global patterns and trends in colorectal cancer incidence and mortality. Gut.

[42] Patton N, Aslam T, Macgillivray T, Pattie A, Deary IJ, Dhillon B (2005). Retinal vascular image analysis as a potential screening tool for cerebrovascular disease: a rationale based on homology between cerebral and retinal microvasculatures. J Anat.

[43] Wong TY, Mitchell P (2007). The eye in hypertension. Lancet.

[44] Targher G, Byrne CD, Lonardo A, Zoppini G, Barbui C (2016). Non-alcoholic fatty liver disease and risk of incident cardiovascular disease: A meta-analysis. J Hepatol.

[45] Migliorisi G, Vella G, Dal Buono A, Gabbiadini R, Busacca A, Loy L, Bezzio C, Vinciguerra P, Armuzzi A (2024). Ophthalmological Manifestations in Inflammatory Bowel Diseases: Keep an Eye on It. Cells.

[46] Lu F, Du Y, Gu Y, Jiao Y, Gu Y, Shen D, Xia Y, Yu J (2025). OCT-based retinal parameters and choroidal vascular indices predicting response to anti-TNF-α therapy in inflammatory bowel disease. Photodiagnosis Photodyn Ther.

[47] Rinella ME, Lazarus JV, Ratziu V, Francque SM, Sanyal AJ, Kanwal F, Romero D, Abdelmalek MF, Anstee QM, Arab JP, Arrese M, Bataller R, Beuers U, Boursier J, Bugianesi E, Byrne CD, Castro Narro GE, Chowdhury A, Cortez-Pinto H, Cryer DR, Cusi K, El-Kassas M, Klein S, Eskridge W, Fan J, Gawrieh S, Guy CD, Harrison SA, Kim SU, Koot BG, Korenjak M, Kowdley KV, Lacaille F, Loomba R, Mitchell-Thain R, Morgan TR, Powell EE, Roden M, Romero-Gómez M, Silva M, Singh SP, Sookoian SC, Spearman CW, Tiniakos D, Valenti L, Vos MB, Wong VW, Xanthakos S, Yilmaz Y, Younossi Z, Hobbs A, Villota-Rivas M, Newsome PN, NAFLD Nomenclature consensus group (2023). A multisociety Delphi consensus statement on new fatty liver disease nomenclature. Hepatology.

[48] European Association for the Study of the Liver, European Association for the Study of Diabetes, European Association for the Study of Obesity (2024). EASL-EASD-EASO Clinical Practice Guidelines on the management of metabolic dysfunction-associated steatotic liver disease (MASLD): Executive Summary. Diabetologia.

[49] Orfanidou M, Polyzos SA (2025). Retinopathy in metabolic dysfunction-associated steatotic liver disease. Medicina (Kaunas).

[50] Oh Y, Song SJ (2025). Association of Steatotic Liver Disease with Retinal Vascular Occlusion: The Influence of Obesity in a Large Health Screening Cohort. Endocrinol Metab (Seoul).

[51] Qian XZ, Hu JY, Zeng YM, Wei H, Wang XY, Chen C, Ge QM, Zou J, Zhou XM, Ling Q, He LQ, Liao X, Shao Y (2025). Retinal microvasculature alteration in patients with acute pancreatitis: an observational OCTA study. Eur J Med Res.

[52] Collins GS, Moons KGM, Dhiman P, Riley RD, Beam AL, Van Calster B, Ghassemi M, Liu X, Reitsma JB, van Smeden M, Boulesteix AL, Camaradou JC, Celi LA, Denaxas S, Denniston AK, Glocker B, Golub RM, Harvey H, Heinze G, Hoffman MM, Kengne AP, Lam E, Lee N, Loder EW, Maier-Hein L, Mateen BA, McCradden MD, Oakden-Rayner L, Ordish J, Parnell R, Rose S, Singh K, Wynants L, Logullo P (2024). TRIPOD+AI statement: updated guidance for reporting clinical prediction models that use regression or machine learning methods. BMJ.

[53] Senterkiewicz M, Szaflarska-Popławska A, Kałużny BJ (2025). Ocular Manifestations of Celiac Disease: Current Evidence and Clinical Implications. Nutrients.

[54] Tasli NG, Gunay BO, Ugurlu A, Eren MA, Aykut M, Esenülkü CM (2025). Optical coherence tomography angiography in non-alcoholic fatty liver disease: is it a disease affecting the microvascular system??. BMC Ophthalmol.

[55] Tejani AS, Klontzas ME, Gatti AA, Mongan JT, Moy L, Park SH, Kahn CE, CLAIM 2024 Update Panel (2024). Checklist for Artificial Intelligence in Medical Imaging (CLAIM): 2024 Update. Radiol Artif Intell.

[56] Sounderajah V, Guni A, Liu X, Collins GS, Karthikesalingam A, Markar SR, Golub RM, Denniston AK, Shetty S, Moher D, Bossuyt PM, Darzi A, Ashrafian H, STARD-AI Steering Committee (2025). The STARD-AI reporting guideline for diagnostic accuracy studies using artificial intelligence. Nat Med.

